# The Successful Dentist

**Published:** 1896-09

**Authors:** C. E. Bentley

**Affiliations:** Chicago, Ill.


ARTICLE VII.

### THE SUCCESSFUL DENTIST.

BY C. E. BENTLEY, C. D. S., CHICAGO, ILL.

It has often occurred to me that amid the din and battle of competition which is so hotly waged among the followers of our profession, in our greed we might dull our sense of the true and the beautiful which so largely characterizes the true success.

The pursuit of the almighty dollar is to-day our supreme vocation—with it as a corollary goes gross materialism. That is to say, wherever and whenever a people subordinate their finer sensibilities at the altar of Mammon, gross materialism follows close on its heels.

The world applauds the money-getter; closes its eyes as to how he gets it; rocks him in the lap of indulgence till some day the very child of its fondling shows its teeth of avariciousness and dehumanizing instincts and a clash ensues. The offspring of its caresses becomes its common enemy, and they stand armed and arrayed against each other. Inordinate selfishness has blinded its sight to the true and the beautiful.

I applaud the man who, by his superior genius, is able to so marshal the forces of nature and so master the intricacies of cause and effect as to wring from nature's labyrinthic hiding-places the treasures to which they so tenaciously cling. But while I applaud the money-getter, I revere the money-spender. The man is the champion of the true and the beautiful who considers himself a trustee of the wealth he has accumulated, using the power his wealth gives him for good and not for evil—spreading sunshine instead of gloom—humanizing instead of dehumanizing.

Men who sacrifice their human interests for the benefit of the common weal; men who give thousands on thousands for institutions which brighten the gloom of ignorance, illume benighted minds, rearing good citizens grounded in the principles of good citizenship; men who have a firm grasp on the living sociological questions of the day and build for the morrow.

To thc student of the affairs of the cociological horizon this tendency on the part of our business men, of our public schools, lends a hopeful sign.

If what I have said of the business man be true, how much more am I to expect of the professional man ?

A storekeeper may sell you a piece of shoddy for wool at the wool price, and has only injured you to the extent of deceiving you by his wilful misrepresentation.

If a physician is called to the bedside where the life of his patient is interested in his care, and he wilfully misrepresents the case or his ability, it would be a grave offense. The injury, unlike the case of the merchant, might be irreparable; a life might go out as a result of his duplicity.

The lines that lead to true success are the same everywhere. For one to be a successful dentist, he must be first enthusiastic, he must be ablaze. "A man with only one idea all aglow with enthusiasm will accomplish more than a ripe scholar with a thousand great thoughts hidden away in pigeon-holes."

Away with your icebergs, though they glisten beautifully. Give us men with warm, genial, inspiring natures; intelligent, but with intense passion; aggressive, daring, venturing their life on success. Such men will find their places somewhere, some how, some time.

A professional man should be cultured. The more liberally educated a man may be, the better dentist he will make. I am here reminded of Prof. Huxley's criticism of the empiricists. Says he, "Indeed I am so narrow minded myself that if I had to choose between two physicians, one who did not know whether a whale is a fish or not, and could not tell gentian from ginger, but did understand the application of the institutes of medicine to his art, while the other, like Talleyrand's doctor, knew a little of everything, even a little physic, with all my love for breadth of culture, I should assuredly consult the latter." But in reality the man who is greedy of what his own art contains is not satisfied with being illy informed of the things outside.

He must have individuality. Show your colors; not offensively, but be not ashamed of your convictions. Are you ashamed of the rottenness that permeates your municipal government? If so, say it, act it, vote it. He should not attend church for the business he could get out of it, but for the benign influence that goes with that attendance to a part of man's nature that has been recognized since man came on the earth.

He should be alive to and in touch with the burning questions of the day, conversant with good literature, keeping space with civilization's progress, and always in the front rank of his profession. It is easy to be a nonentity and sink in the grave of oblivion, but it needs courage to be the representative of something useful and aggressive.

He must be physically sound, without which he will be unable to bring the serviceable attributes and the fertile brain so necessary in the successful practice of his profession.

His manner should be good; his demeanor kind and gentle, indicating the inner worth.

He must be morally sound. This is the keynote to the symphony, the compass of the ship, the main path to the broad highway of success. The moral safety of the patients entrusted to his care must be an assurance beyond peradventure; like Cæsar's wife, he must be above suspicion. He must collect his bills and pay his debts.—*Dental Review.*

